# Blockchain-Based Secure Authentication with Improved Performance for Fog Computing

**DOI:** 10.3390/s22228969

**Published:** 2022-11-19

**Authors:** Otuekong Umoren, Raman Singh, Shahid Awan, Zeeshan Pervez, Keshav Dahal

**Affiliations:** School of Computing, Engineering and Physical Sciences, University of the West of Scotland, Paisley PA1 2BE, UK

**Keywords:** authentication, Neo blockchain, fog computing, internet of things, blockchain technology, cloud computing, smart contracts

## Abstract

Advancement in the Internet of Things (IoT) and cloud computing has escalated the number of connected edge devices in a smart city environment. Having billions more devices has contributed to security concerns, and an attack-proof authentication mechanism is the need of the hour to sustain the IoT environment. Securing all devices could be a huge task and require lots of computational power, and can be a bottleneck for devices with fewer computational resources. To improve the authentication mechanism, many researchers have proposed decentralized applications such as blockchain technology for securing fog and IoT environments. Ethereum is considered a popular blockchain platform and is used by researchers to implement the authentication mechanism due to its programable smart contract. In this research, we proposed a secure authentication mechanism with improved performance. Neo blockchain is a platform that has properties that can provide improved security and faster execution. The research utilizes the intrinsic properties of Neo blockchain to develop a secure authentication mechanism. The proposed authentication mechanism is compared with the existing algorithms and shows that the proposed mechanism is 20 to 90 per cent faster in execution time and has over 30 to 70 per cent decrease in registration and authentication when compared to existing methods.

## 1. Introduction

Blockchain technology has developed over the years and made room for decentralised applications to be incorporated or combined with other technologies. Blockchain uses a distributed ledger maintained through various nodes connected through networks; these nodes are also responsible for the communication and recording of transactions [1]. Blockchain’s distributed and decentralised ledger has revolutionized security with its secure data storage mechanism, which involves the registration of transactions with immutable cryptographic signatures [2]. With the fast progress and technological improvements in fog computing, the Internet of Things (IoT), and cloud computing, more and more edge devices are added to the edge layer of connected services. These smart devices on this edge layer gather and transmit sensitive data to fog devices over the cloud environment. This arrangement puts data at risk and increases the chances of security breaches [3]. This vulnerability has made the security of smart and IoT devices a major problem in IoT, fog, and cloud computing. Internet of Things has an important role in cloud services, some of which is the collection of data through sensors and the transmission of data through networks and the internet. These sensors and devices communicate with each other through networks. Authentication is considered a major security concern in IoT, mainly user authentication and the authentication of IoT devices [4]. The authors in [5] highlighted the properties of blockchains, including immutability, decentralisation, security and consensus, and the benefit of integrating blockchain technology to improve big data security and privacy, improve data integrity, real-time data analytics, enhance data sharing, and quality of big data. Various blockchains platform, such as Multichain, Ethereum, Bitcoin, Neo blockchain, etc., have been introduced over time, with  unique advantages over the others. Different blockchain platforms operate on different consensus protocols and guarantee security along with a different level of scalability [6]. Smart or edge devices collect and generate large quantities of data almost instantly; the data gathered gets passed on to the cloud through multiple IoT devices connected to networks, occasionally being the source of overloads on the network [7]. The introduction of fog computing guarantees a decentralised and distributed computing environment involving and utilizing multiple fog nodes or devices engaging in different or multiple locations. The fog environment functions between the edge and cloud layer, and the fog nodes manage data processing seamlessly. This helps overcome computing limitations in cloud and IoT or edge devices and provides an improved solution for the improvement of services that depend on cloud computing. Thus, the data collected or gathered by edge devices are processed swiftly and transferred to the cloud, hence achieving its purpose of providing edge and IoT devices with cloud computing services to limit network traffic or communication with the cloud [8].

Generally, authentication, authorisation, and access control systems guarantee the security of all data types collected and saved on cloud servers. Secure authentication is provided through methods of restriction and access control to data and various services. However, a lot of authentication, authorisation, and access control systems depend on centralised storage or a database and the use of trusted third parties, which makes them at risk of various breaches or attacks [9]. The distinctive features of fog computing, namely the ability to compute data and the distributed nature of fog devices and the properties of blockchain technology, such as the smart contract, decentralised ledger, and authentication systems, would take advantage of these features and properties and ensure secure authentication in a cloud and IoT environment.

Some notable cyberattacks include the RockYou2021, which is considered the largest password collection leakage. This attack was about eight billion entries of passwords [10]. In April 2022, the Romanian government websites became targets of a cyberattack; this involved the use of distributed denial of service attacks with attempts to cause an overload on systems by sending multiple requests to targets from many origins. These attacks were targeted at the websites of the defence ministry, the border police, a financial institution, and a railway company [11]. Equifax, in September 2017, announced at the time that it had been a victim of a cyberattack that led to the compromise of the file containing fifteen million two hundred thousand United Kingdom records dating from 2011 to 2016; these records included personal information of customers and duplicates of data [12]. Other recent cyberattacks include A DDoS attack which forced the Port of London Authority offline in May 2022 and the attack on a stock trading platform Robinhood in November 2021, which allowed the hacker access to the personal information of about seven million users; these data included names, email addresses, date of birth, and zip codes [13]. Security in IoT is critical, mostly due to the wide range of security threats on networks. Additionally, there are  insecure practices of individuals, users, groups, or organisations who lack or have limited resources or knowledge of practices to protect their IoT systems or architecture. Despite the limited resources of some IoT devices or edge devices, they can still be exploited through malware [14]. An example of an attack through an IoT system is the attack on Dyn servers on 21 October 2016; this server controlled most domain name system infrastructure on the internet, leaving several websites, including Netflix, CNN, Twitter, and others, down in the United States and Europe. This distributed denial of service attack was carried out using a network of IoT devices infected with a mirai botnet coordinated to flood the server with traffic till it collapsed [15]. In this paper, we propose an improvement in a decentralised fog computing authentication scheme utilizing Neo blockchain technology. This improved system utilizes data classes, which include username, password, and Neo wallet ID during the process of user registration and user authentication. The Neo blockchain properties, including its decentralised ledger, smart contract, and consensus protocol, improve user authentication by utilizing the decentralised storage and peer-to-peer communication between fog nodes. The proposed system is an improvement from our previous research work [16], and it achieves fast, secure, and improved authentication and communication.

This research paper’s major contributions are as follows:

1. A security mechanism with improved performance is presented for a fog computing environment.
2. The proposed system is designed to execute user registration and authentication along with the capability of providing data security in a decentralised environment.
3. The delegated Byzantine Fault Tolerant (dBFT) shows improvement over proof of work (POW) and proof of stake (POS).
4. The proposed system offers more reliable security and speed when compared to other existing blockchain solutions.

## 2. Related Works

Several research works that utilize blockchain technology and its unique characteristics have been proposed by different researchers. These research methodologies are based on fog computing, auctions, authentication, IoT, weather-based insurance, and other decentralised systems. A detailed review of schemes has been carried out on the blockchain, authentication, fog computing, IoT, and cloud computing for a better understanding of the proposed scheme. In [3], researchers developed a blockchain-based IoT identity authentication system. The system uses four steps to verify the identity of the user and devices. By following these steps, the user and device generate the key using the ellipse encryption algorithm; the hash value of this key serves as the system’s authentication ID. Through IoT devices, the account information is registered into the block. Upon sending transaction information to the smart contract and receiving read permission from the device, the contract is established, and contact between the device and the user is created. An authentication system was proposed in 2018 [17]. This proposed system assigned unique identities for every single device and had these devices’ identities recorded on the blockchain to enable them to identify each other with a centralised database. Besides data protection, this system detects changes in data by hashing significant data into the blockchain. Intending to improve the authentication efficiency, ref. [18] proposed a distributed and trusted authentication system based on blockchain and edge computing. There are three layers in this system: the physical network layer, the blockchain edge layer, and the blockchain network layer. As a result of this guarantee of trusted authentication, terminal-to-terminal traceability was achieved. Lau et al. [4] addresses the issue of authentication in IoT networks, and the Authenticated Devices Configuration Protocol (ADCP) was developed. By using blockchain technology, IoT devices can be digitally identified and authenticated before they join a network by creating digital identification and authentication codes. Researchers in [19] implemented a blockchain technology-based system for a cellular mobile network using smart contracts. Mobile network cores can now operate in a secure distributed environment, while smart contracts allow operators to handle their transactions in a self-organizing network. To solve the encryption, decryption, signature, and verification issues of blockchains, a heterogeneous alliance network was established to develop an identity authentication system based on domestic, commercial cryptography combined with blockchain. This was one of the distributed methods for creating private and public keys for key security [20]. Gong et al. [21] introduced a device recognition model for blockchain-based identity authentication that utilised a novel feature selection method for device traffic flow to simplify the recording of transactions during authentication on a blockchain network without replacing or swapping existing hardware or software. Zhaofeng et al. [22] A blockchain-based decentralisation authentication protocol called BlockAuth was designed to provide secure registration and authentication. Their scheme provided a decentralised authentication system with strong fault tolerance, and high-level security, as well as blockchain consensus. Several authentication methods can be used with this system, including passwords, certificates, tokens, and biometrics. Kalaria et al. [23] As a result of fog computing, a mutual authentication scheme based on one-way hash functions and Elliptic Curve Cryptography has been proposed. This scheme provides security and protection against cyber-attacks to the devices and entities that interact with it. Their scheme achieved mutual authentication in the fog computing environment, but immutability problems persisted. The issue of trust inherited by edge computing was addressed through a scheme [24]. As a result, problems were identified with registering and authenticating servers through trusted entities, as well as challenges posed by a single point of failure enabling threats to exploit the network. Through the elimination of public trusted entities within their network framework, they were able to overcome challenges and difficulties associated with implementing decentralised platforms through permissioned blockchain technology. This scheme allowed authenticated users to access services without sign-ins at all service providers. As a result of using a single authentication mechanism, the system is vulnerable to attacks due to its single authentication mechanism.

In [25], a decentralised access control and authentication mechanism for lightweight IoT devices were proposed. Based on fog computing and blockchain technology, this mechanism utilised blockchain cryptographic properties and a decentralised nature, along with fog computing’s ability to solve latency issues. An authentication scheme based on blockchain-enabled fog nodes and Ethereum smart contracts was proposed to provide access to IoT devices and authenticate users. This approach enables the system to scale by utilizing fog nodes to perform computing tasks [26]. The authors of [27] presented a method for improving mutual authentication that incorporates multiple factors, a challenge-response function, a modifiable response time, and the current time. As a result of fog and cloud computing, methods for regulating these factors could be established. There is a proposed model of an IoT network security network using blockchain technology [28], which incorporates blockchain technology and addresses the difficulties of deploying a blockchain in IoT networks by using genetic algorithms and particle swarm optimisation to dive the network into multilayer decentralised systems called K-unknown clusters. In [29], the authors presented a blockchain-based solution with the aim of improving the privacy and security of virtual circuit-based device data. Their model was implemented in an IoT-based application in a virtual vehicle monitoring system. This system stored information about vehicle reaction, authentication, and integrity in the cloud platform and further stored the data on a public Ethereum blockchain to enable smooth transactions.

FogHA [30] used lightweight cryptographic primitives and fog nodes and proposed an anonymous handover authentication scheme that can offer key management and mutual authentication between the fog node and a mobile device by eliminating unnecessary authentication messages. There were features such as untraceability, anonymity, and low latency that made this scheme resistant to attacks from insiders. Adversaries could utilize the untraceability and anonymity to gain an advantage in this scheme, executing attacks without being detected by the system. As outlined in [31], an identity management mechanism was developed so that devices would remain anonymous and session keys would be securely negotiated through authentication. As a result, trust was not restricted to a single domain and blockchain technology was used for authentication. As a result, it could be exploited by rough devices or adversaries to authenticate devices on different administrative domains without knowing their identities. Bubbles of trust are proposed by [32] for the identification and authentication of IoT devices using a decentralised system. By utilizing blockchain technology, it can secure data integrity and availability through the use of virtual zones and servers, and devices within the virtual zones can independently verify and identify each other. Because of their importance to the system and potential attack targets, these virtual zones must be secured.

As discussed in this section, the existing schemes and systems focus on authentication in IoT and fog environments and integrate blockchain technology to enhance security and decentralisation. However, most of the schemes utilised the Ethereum blockchain while some depended on centralised databases or central authorities, which have limitations. We propose a decentralised fog and IoT authentication system based on Neo blockchain technology. This is an improvement from our previous system [16] and aims to address the limitations in decentralised authentication systems based on the Ethereum blockchain. Table 1 summarizes the existing works and the blockchains used in their schemes.

## 3. The Proposed Methodology

In this section, the research methodology, along with the experimental setup and implementation of the proposed Neo blockchain-based authentication system, is discussed. The proposed methodology also consists of a few assumptions as given by the researchers in [8] and listed below:

- Through multiple networks and the internet, multiple mobiles and non-mobile devices are connected to the fog computing environment.
- Blockchain nodes are accessible to authorized users and devices.
- To serve as nodes or servers and host the blockchain, fog devices need to meet certain requirements.
- It is necessary for smart contracts to perform tasks that are programmed.
- A task can be performed by a node without relying on another node.

**Figure 1:**
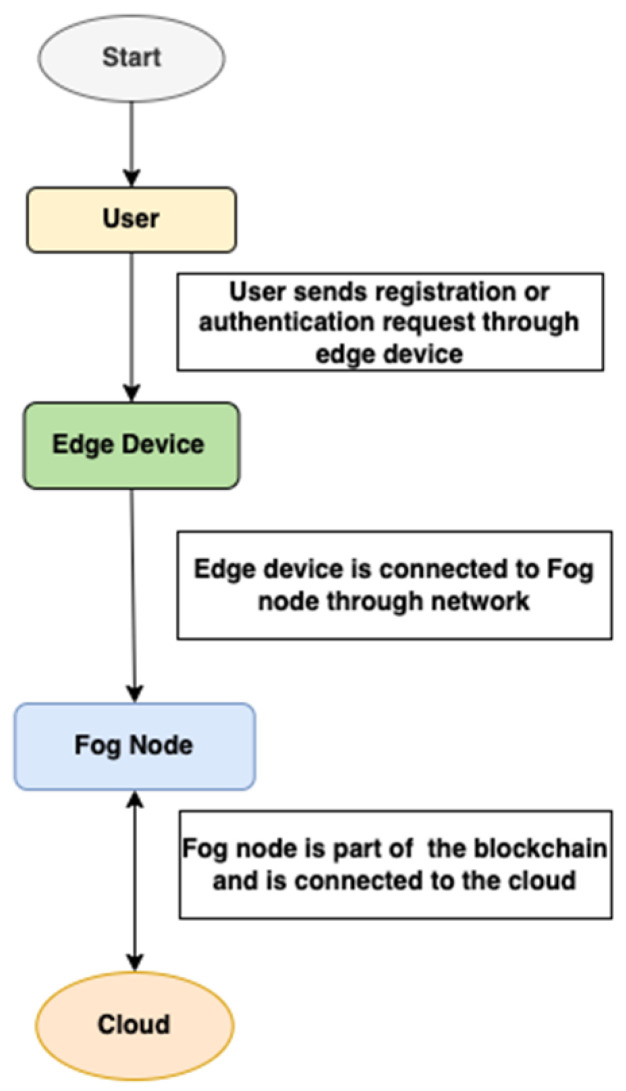
Flowchart of the overall architecture of the proposed system.

The proposed system utilizes Neo blockchain, smart contracts, and ledgers to provide decentralised authentication for IoT and fog computing. The fog nodes execute blockchain tasks and keep a copy of smart contracts and decentralised ledgers. The overall architecture of the proposed scheme is given in Figure 1. The system starts with the procedure where a user sends the authentication request through the user or edge device. This edge device is connected to the fog device through a network. The fog device is connected to blockchain nodes and has access to the smart contract execution module.

### 3.1. Advantages of Neo Blockchain over Ethereum Blockchain

The Neo blockchain has some properties that give it advantages over the Ethereum blockchain; these advantages include [40]:

- Consensus mechanism: delegated Byzantine Fault Tolerant (dBFT), which is considered an improvement over Ethereum’s Proof-of-Stake and is more energy efficient.
- Hard-fork proof due to delegated Byzantine Fault Tolerant.
- Speed: Executes 10,000 transactions per second compared to Ethereum’s 15 transactions per second.
- Quantum computer proof using Neo QS.

Various aspects of different components of the proposed system are explained as given below.

### 3.2. System Architecture

**Figure 2:**
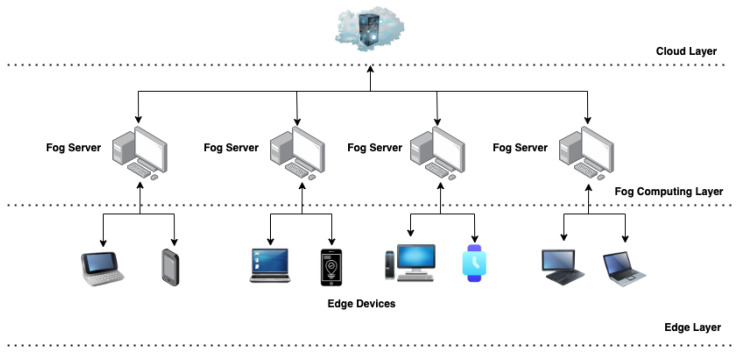
Authentication system built on Neo blockchain and fog computing.

The proposed decentralised authentication system is described in this section, along with the tasks they complete. The complex architecture of the proposed system is shown in Figure 2. It shows the authentication system built on the Neo blockchain using edge and fog devices. The components involved in this architecture are described below:

#### 3.2.1. Neo Smart Contract

Authentication and registration tasks are executed by the contract in this system. The contract requires data such as *Username*, *Password*, and *UserNeoAddress* during registration and authentication and as the user interacts with the system thereafter.

#### 3.2.2. Fog Node

There are several fog nodes in the network that act as blockchain nodes, each having a copy of the *Blockchain*, *Ledger*, and *SmartContract*. A *Blockchain* registration or authentication transaction through a fog node updates the ledger for the *Blockchain* transactions. In order to be able to host or be a part of *Blockchain*, these fog devices or servers must meet the required specifications.

#### 3.2.3. Users

For authentication and registration to be successful, the user must provide valid data and perform a series of tasks. A valid *Username*, *Password*, and *UserNeoAddress* must be provided for these tasks.

#### 3.2.4. Edge Devices

During registration and authentication, these are the user devices with insufficient resources to host the *blockchain*.

#### 3.2.5. Cloud

Data are the main characteristics of the cloud, which is often described as a large storage unit with the ability to host, store, and compute data. Upon receiving data generated by IoT, edge, and fog devices, this cloud server processes it for analysis and processing.

### 3.3. Proposed System Working

The working of the proposed authentication system is outlined in this subsection.

#### 3.3.1. Initialisation

As part of the registration process, a new user must apply all factors necessary for the authentication system to work. These parameters initialised by the Neo blockchain are valid *UserNeoAdr* (with Neo Gas), *Username*, and *Password*.

#### 3.3.2. User Registration

**Figure 3:**
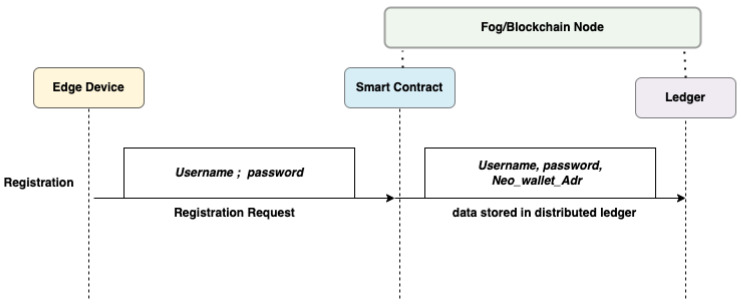
Flow diagram for User Registration.

In order to complete the registration phase, a new user sends a registration request and is asked to provide *Username* and *Password*, as well as *UserNeoAdr*, to *BlockChainNet*; the data are transferred through the *SmartContract* and stored in the *Ledger*. The *Blockchain* identifies the *User* as a valid *User*, and the *Ledger* stores the data provided by the *User* on the *Blockchain*. Figure 3 shows the user registration process.

#### 3.3.3. User Authentication

**Figure 4:**
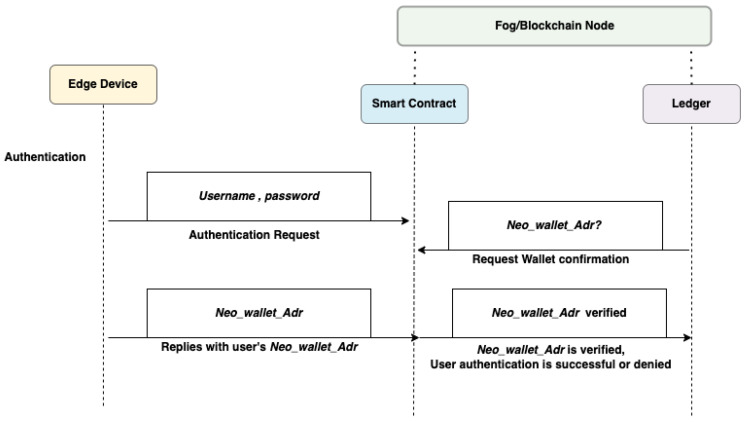
Flow diagram for User Authentication.

When the authentication phase begins, the *User* sends an authentication request with the *Username* and *Password*, and the *User* must also submit a valid *UserNeoAddress*. As part of the *Blockchain*, the *User* is verified through the *SmartContract* and *Ledger*. Data presented by the *User* determines whether the authentication attempt is successful. Valid data are required from the *User* for authentication to succeed. A representation of user authentication can be found in Figure 4. In order to authenticate successfully, the *User* must provide data that matches the parameters the system requires. If successful authentication is not achieved, the *User* gets another opportunity.

### 3.4. Implementation

The purpose of this section is to introduce the algorithms for registering a user in the proposed system, as well as identifying and authenticating the user. A pseudocode for registering a user using a *Password* and *Username* is included in Algorithm 1. In addition, a new *UserNeoAdr* is created for the new *User*, and all the new *User* data are stored in the *BlockChainNet*, and the new *User* is registered successfully.
**Algorithm 1** Pseudocode showing steps to register users1:**function** new user registration request(Username, password)2:    **if** (Username, password = True) **then**3:        NewUser(Username, password, UserNeoAdr = True)4:        BlockchainUser (new user data stored on the Blockchain using the smart contracts)5:        Log (New user registered)6:        **return** true7:        else8:    **end if**9:**end function**

**Figure 5:**
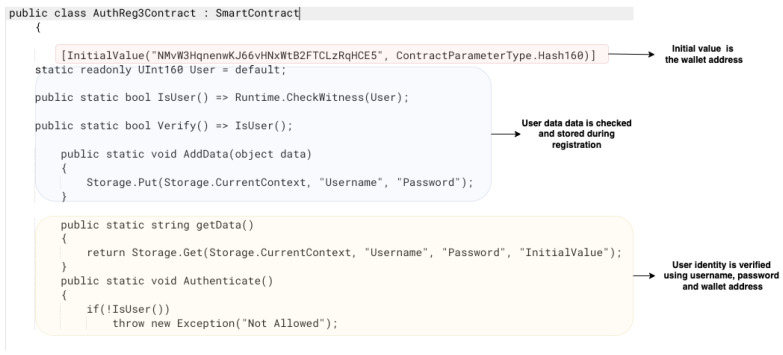
Smart contract for user registration and authentication.

A pseudocode for user authentication requests based on the data *Username*, *Password*, and *UserNeoAdr* of a valid and existing *User* is included in Algorithm 2. By validating *User* data submitted for authentication, the *BlockchainNet* determines whether authentication will be successful or termed as unsuccessful. A brief description of functions in the smart contract can be found in Figure 5.
**Algorithm 2** Pseudocode showing steps to authenticate users1:**function** user authentication request(Username, password, UserNeoAdr)2:    **if** (received authentication request from user = True) **then**3:        valid user data and NeoAdr–stored User data in blockchain through smart contract4:        **if** (User data and NeoAdr is user data stored on the Blockchain = true) **then**5:           Authentication successful6:           Log (“authentication successful”)7:           **return** true8:           else9:           Log (“Authentication failed”) **return** false10:        **end if**11:    **end if**12:**end function**

## 4. Result Analysis and Discussions

To assess the performance of the proposed system, several experiments have been conducted to evaluate the method. Using the C sharp programming language, the proposed system is implemented with Neo smart contracts. This smart contract is created and tested using the Neo blockchain toolkit in Microsoft visual studio code [41]. The Microsoft visual studio code environment provides the environment for working with the Neo blockchain toolkit, which is used to create private networks, multiple users, and environments for Neo blockchain. Furthermore, this environment allows you to create, deploy, test, and debug smart contracts. The Neo blockchain network is simulated in the Microsoft visual studio code environment, and the layout of the system is then simulated in Cisco Packet Tracer. This experiment was conducted using Microsoft Visual Studio code for its ability to allow smart contracts to be developed, created, implemented, and deployed in a virtual Neo blockchain environment. A virtual IoT environment and virtual network can be created and simulated using Cisco Packet Tracer’s network simulation environment [42]. Each virtual Neo blockchain account had a wallet filled with Neo gas and represented a unique user created using the Neo blockchain toolkit in Microsoft Visual Studio Code. A multiple-test registration and authentication process is implemented, and the metrics for each test are analysed. The metrics are used to collect data, including registration and authentication data. In the present study, the emphasis is given to secure authentication, and registration/authentication time will be analysed in future research. A working IoT and fog environment has been simulated using Cisco Packet Tracer, using nodes and edge devices to create a virtual IoT and fog network. The performance and simulation results are included in the subsections below.

### 4.1. Performance Metrics and Results

In this experiment, several metrics given below are taken into consideration, including the user registration gas, the user authentication gas, and the elapsed time. It is necessary to compare and evaluate the proposed scheme with existing methods [8,16,26]; these existing methods were chosen because they also utilised the blockchain for authentication or security in fog computing.

- Registration gas: The amount of Neo gas used during registration.
- Authentication gas: The amount of Neo gas used during authentication.
- Elapsed time: Time required for registration and authentication.

#### Network Simulation in Cisco Packet Tracer

**Figure 6:**
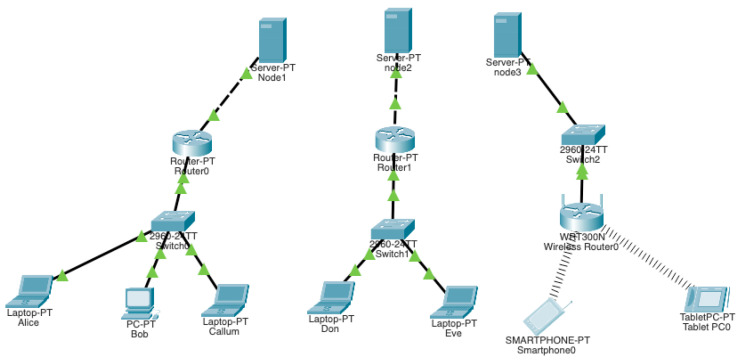
Simulation of the fog computing environment in the Cisco packet tracer.

The IoT and fog environment has been simulated in Cisco Packet Tracer [42]; this simulation involved a virtual network of fog servers (nodes) and user edge devices configured and connected in networks to simulate a real-world networking architecture and run tests using various packets and protocols. Multiple tests are executed over wired and wireless networks where packets are sent from edge devices to the fog server, from edge devices to other edge devices, and from the fog server to other fog servers. The system architecture created in the Cisco Packet Tracer is displayed in Figure 6. In the architecture, nodes 1 and 2 are connected to edge devices (laptops and PC) through routers and switches, these are wired connections, while node 3 is connected to edge devices (smartphone and tablet computers) through a switch and wireless router.

### 4.2. Performance Evaluation

This section aims to compare the performance and results of the proposed system with those of the existing methods [8,16,26]. Using the metrics of registration, authentication, and elapsed time, the proposed and existing methods have been displayed and can be identified in the charts in this section.

The scheme [26] was designed for registration and authentication; this used blockchain-enabled nodes to execute registration and authentication. Ref. [8] is a decentralised mutual authentication, this used blockchain for device registration, authentication, and location validation. Ref. [16] was designed for user registration and authentication in a decentralised secure fog computing, it utilised blockchain smart contracts to register and authenticate users. The proposed system performs faster than the existing methods [8,26], as indicated by the elapsed time. In an evaluation, it is observed that it is scalable with increasing registrations and authentication requests with a slight increase in elapsed time and registration gas. Four groups of user devices are used for this experiment, 5, 10, 15, and 20 devices, to compare with existing methods. For proper comparison and evaluation of results, experimental results from existing methods [8,16,26], which were executed on the Ethereum blockchain, have been converted from ether to neo gas. This conversion was executed with the current rate. The simulation results for registration and authentication are included below.

#### 4.2.1. Registration Simulation

**Figure 7:**
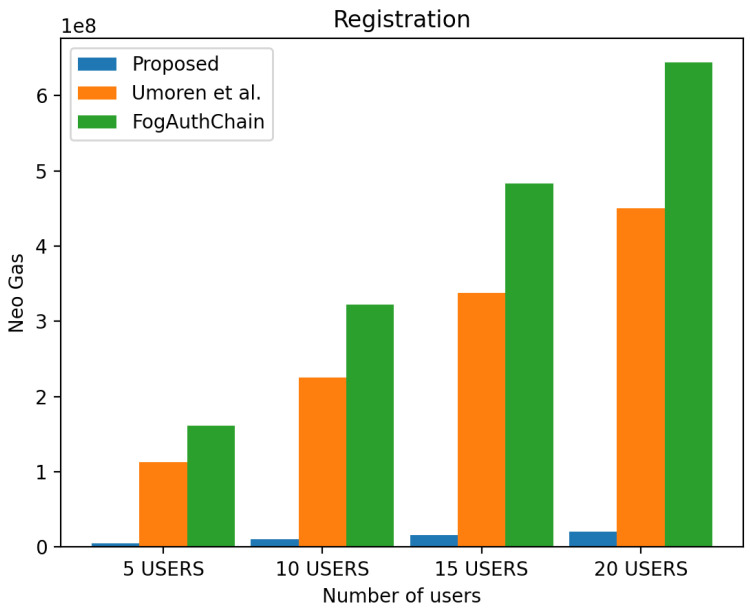
Evaluation of experimental results on registration gas for the proposed system and the existing system [8,16].

We observed over a 70 per cent decrease in registration cost when compared to existing methods, as shown in Figure 7. It is evident from Figure 7 that users in all groups are experiencing a gas decrease compared to the existing methods [8,16].

#### 4.2.2. Authentication Simulation

**Figure 8:**
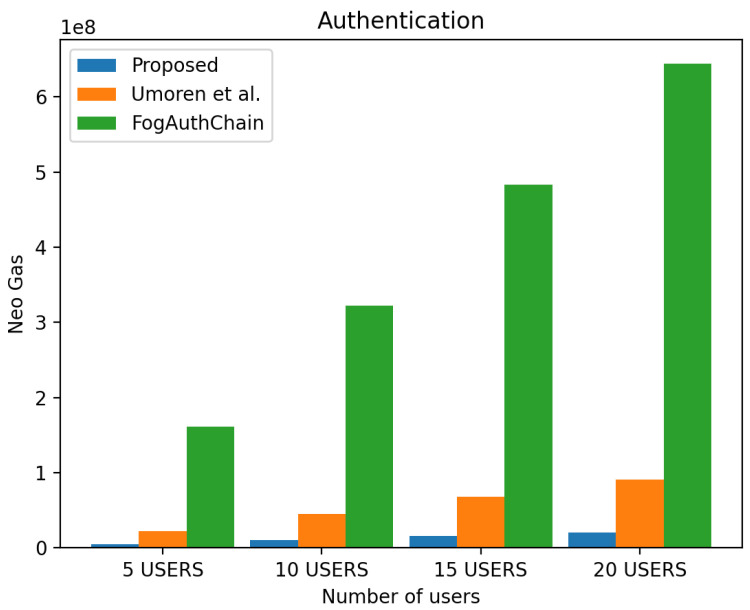
Evaluation ofexperimental results on authentication gas for the proposed system and the existing system [8,16].

The proposed system generated over an 80 per cent decrease in authentication gas compared to the existing system [8] and over a 30 per cent decrease when compared to [16], as shown in Figure 8. Based on a comparison of the metrics from [8,16] to the metrics from the user groups Figure 8, it is found that there is an overall decrease in Neo gas consumption in all user groups.

#### 4.2.3. Elapsed Time

**Figure 9:**
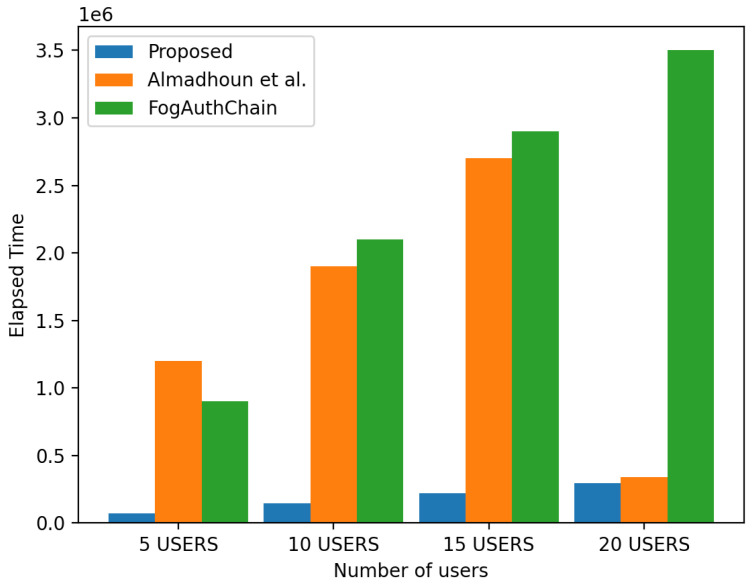
Simulation results showing the time required to send packets [8,26].

Figure 9 displays the elapsed time for registration; the proposed system used less time compared to existing methods [8,26]; this decrease is up to 10 per cent. Figure 9 displays the elapsed time for authentication. Comparing the results of [8,26] for five to ten users, a 20 per cent decrease is observed. In comparison to the existing method [26], times are recorded as higher for the group of 15 and 20 users.

### 4.3. Discussion

Neo blockchain is reviewed in this section to illustrate its advantages over other blockchains, such as Ethereum, and to show potential vulnerabilities. Security and secure authentication are common issues in IoT and fog computing, which are attributed to factors such as the ever-growing number of sensors and IoT devices, their ability to gather and manage data, and their connection to networks that require security [43]. The decentralised nature of the fog computing environment makes it beneficial for IoT and cloud computing; this ensures the computing and processing of data on the fog level, hereby offloading computation on the cloud level and reducing the time required for processing, transfer, and computation. The introduction of Neo blockchain in IoT and fog computing environments mitigates attacks and gives a solution to the problems surrounding secure authentication and security in IoT environments. This ensures secure authentication and mitigates attacks that target fog and cloud computing environments, such as authentication attacks, denial of service, sniffing, man-in-the-middle attack, distributed denial of service, side channel attacks, and so on. Data collection and transmission are the responsibilities of edge devices, sensors, and other resource-limited devices within cloud computing environments. Fog computing is developed to meet the demands of resource-constrained edge devices and sensors, which include secure authentication. Nonetheless, fog computing is not without limitations, some of which are privacy and security challenges inherited from cloud computing [44]. The introduction of blockchain technology offers a solution to these issues. Through the use of blockchain technology, security is provided through the storage and validation of data and records in distributed databases, which mitigates denial of service attacks, man-in-the-middle attacks, spoofing, and other authentication attacks. The blockchain provides security for sensitive data transmitted from one node to another due to its unique characteristics, such as privacy, anonymity, and mutual authentication. Multiple attacks are resistant to the system due to the distributed ledger and validator nodes. Because sensors and edge devices have limited computing and networking capabilities, integrating blockchain technology with IoT and fog computing presents challenges. This includes the limited ability of these sensors and edge devices to execute registration, authentication, or recognition of other devices on the network. Thus, blockchain-enabled fog nodes are tasked with the execution of these tasks while using these edge devices as a module for data collection and transfer. In general, IoT, fog, and cloud computing environments can benefit from the unique characteristics and advantages of Neo blockchain technology. Some advantages of the Neo blockchain over the Ethereum blockchain include speed, considering Neo’s transaction speed is 10,000 transactions per second, compared to Ethereum’s 10 to 15 transactions per second, and Neo blockchain’s instant block confirmation time compared to Ethereum’s six-minute block confirmation time [45]. In a nutshell, the delegated Byzantine Fault Tolerant (dBFT) of Neo is considered an improvement over proof-of-stake (POS) and proof-of-work (POW) because it is energy-efficient, makes Neo hard-fork proof, and provides a balance between scalability, security, and performance [46]. Ethereum’s relatively slow speed is due to its proof-of-work mechanism, which is a lot slower than the Neo blockchain. The first mover is also a disadvantage of Ethereum, with more learning from the limitations to build a better network [47]. With blockchain as the foundation for the system, it can be fully scalable, able to scale to multiple IoT devices, and is still secure as a result.

## 5. Conclusions

In this paper, a Neo blockchain smart contract is created and implemented to address secure authentication and other limitations in an IoT and fog computing environment. This included scalability, immutability, and secure authentication of fog devices in a decentralised fog computing environment. The present research also aims to improve our previous work, which utilised an Ethereum smart contract to provide a decentralised secure fog computing environment. The comparison has shown that with the advantages of the Neo blockchain, performance, speed, and security have improved over the Ethereum blockchain. The experiment’s results display the execution time for the task is lower; in addition, the registration and authentication cost, when compared to existing methods [8,16,26], is reduced by 65 per cent. The resulting evaluation shows that the proposed system improves the cost and speed when compared to existing methods.

## Figures and Tables

**Table 1:** Analysing existing work based on the type of blockchain used, consensus mechanism, hard-fork proof, speed, quantum computer proof, and distributed model. (NA = Not Mentioned).

Work	Blockchain	Concensus Mechanism	Hard-Fork Proof	Speed	Quantum Computer Proof	Distributed Model
FogAuthChain [8]	Ethereum	POW	No	Slow	No	Yes
FogHA [30]	NA	NA	NA	NA	NA	Yes
Blockchain meets IoT [33]	Ethereum	POW	No	Slow	No	Yes
B. Gupta [34]	NA	NA	NA	NA	NA	Yes
Khalid et al. [25]	Ethereum	POW	No	Slow	No	Yes
Kalaria et al. [23]	Ethereum	POW	No	Slow	No	Yes
Dechain [24]	NA	NA	NA	NA	NA	Yes
Meng et al. [31]	NA	NA	NA	NA	NA	Yes
Chow and Ma [35]	NA	NA	NA	NA	NA	No
Bubble of trust [32]	Ethereum	POS	No	Slow	No	Yes
AuthCODE [36]	NA	NA	NA	NA	NA	No
Almadhoun et al. [26]	Ethereum	POS	No	Slow	No	Yes
Leandrloffi et al. [27]	NA	NA	NA	NA	NA	No
Masfog [37]	Ethereum	NA	No	NA	No	Yes
FogBus [7]	NA	NA	NA	NA	NA	Yes
DA-SADA [38]	NA	NA	NA	NA	NA	Yes
AttriChain [39]	Ethereum	POW	No	Slow	No	Yes
Umoren et al. [16]	Ethereum	POS	No	Slow	No	Yes
Our Proposed work	Neo	dBFT	Yes	Fast	Yes	Yes

## Abbreviations

The following abbreviations are used in this manuscript:

*Usermail*	User email address
*User*	User
*Username*	User name
*passowrd*	User password
*NeoAdr*	Neo address
*UserNeoAdr*	User Neo address
*FogNode*	Fog Node
*EdgeDev*	Edge Device
*Cloud*	Cloud
*SmartContract*	Smart Contract
*Blockchain*	Blockchain
*BlockChainNet*	Blockchain Network
*Ledger*	Ledger
